# Supplementary material for: Virtual alignment of pathology image series for multi-gigapixel whole slide images
Source: Nat Commun. 2023 Jul 26;14:4502. doi: 10.1038/s41467-023-40218-9 (PMC10372014; doi:10.1038/s41467-023-40218-9)
Supplement: Supplementary file 1 — Supplementary Information [file 41467_2023_40218_MOESM1_ESM.pdf]

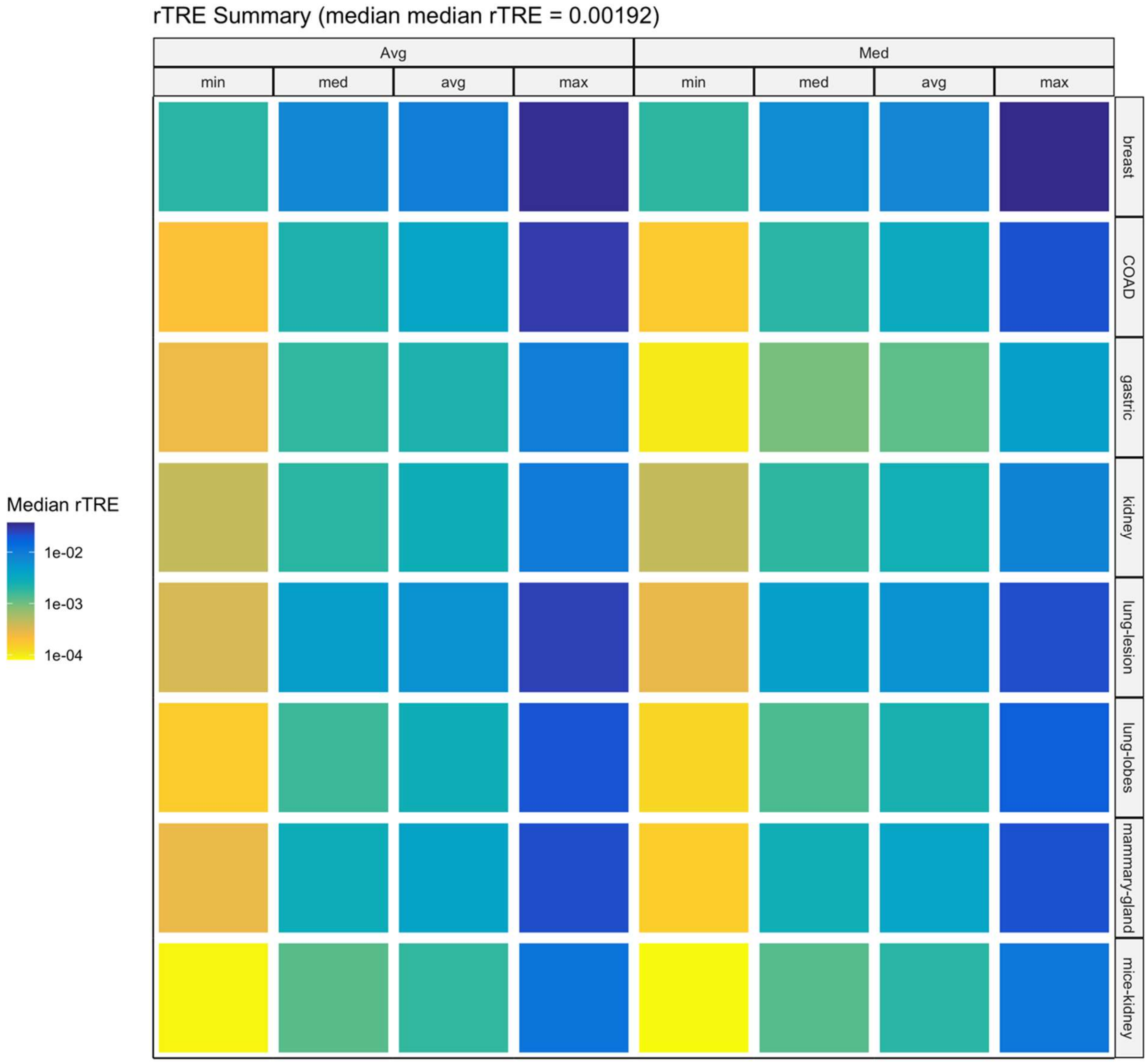

Supplemental Figure 1 Detailed summary of the VALIS' default performance using the Automatic Non-rigid Histological Image Registration (ANHIR) Grand Challenge dataset. Each minor column is a summary of each tissue's median rTRE values, with min=minimum, med=median, avg=average, and max=maximum. The major column "Avg" is the average of the summary statistics, while "Med" is the median of the summary statistics.

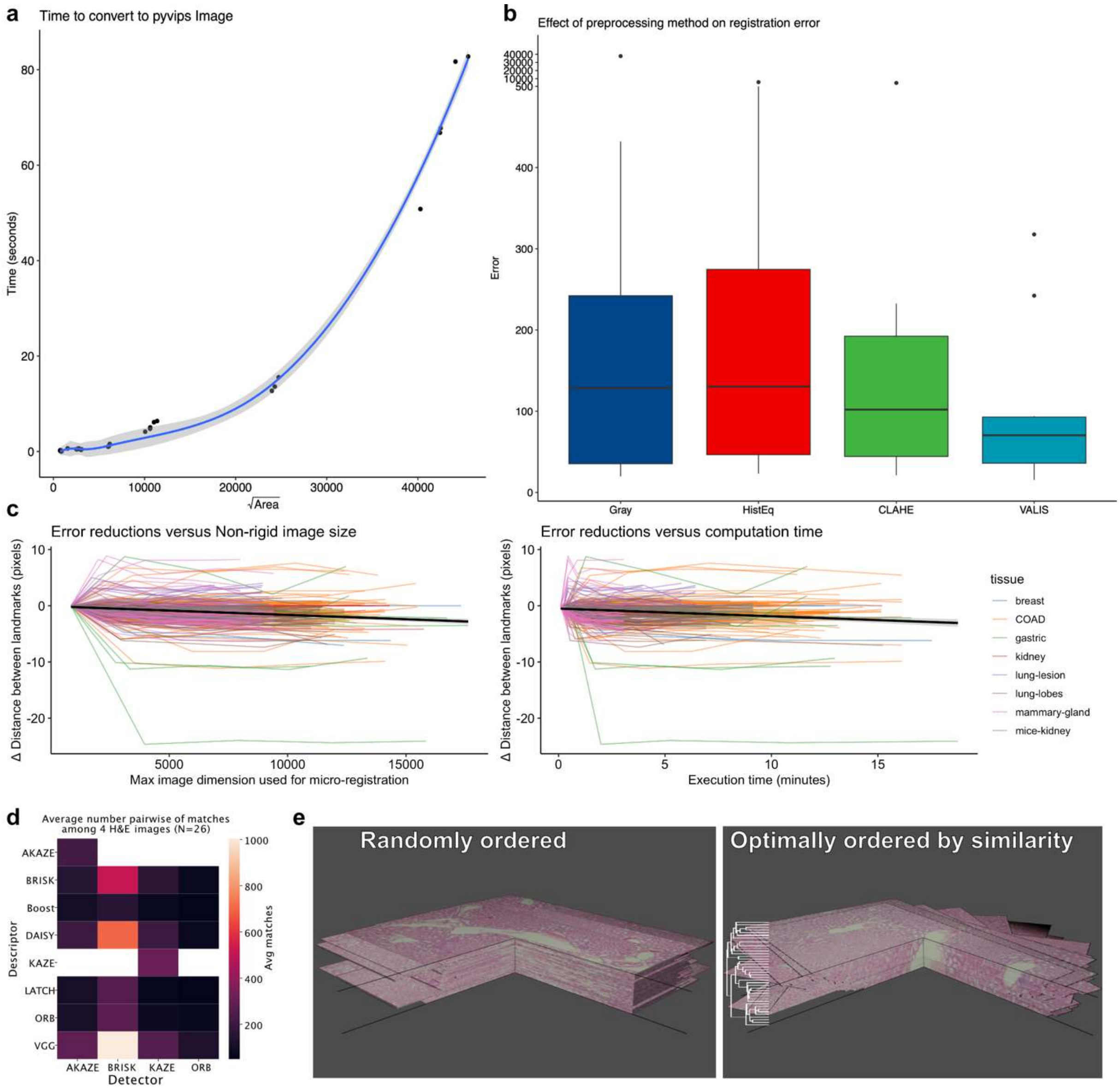

Supplemental Figure 2 Performance benchmarking. a) Time (seconds) required to convert N=57 WSI to pyvips images by stitching together tiles read by Bio-formats. The x-axis is the square root of image area, i.e. 40,000 represents an 40,000 x 40,000 pixel image. During registration, small images are used so the conversion is fast. Blue line is the line of best fit, while ribbon edges indicate 95% confidence intervals. **b)** Effect of pre-processing method on registration error. “Grey” refers to grayscale conversion, “HistEq” refers to global histogram equalization of the grayscale image, “CLAHE” refers to contrast limited adaptive histogram equalization on the grayscale image, and “VALIS” refers the default method used by VALIS to preprocess images. Benchmarking was performed on N=12 unique image pairs (6 DCIS pairs, 4 glioblastoma pairs, and 2 lung adenocarcinoma pairs). In each box, the center line indicates the median, the top and bottom indicate the 75th and 25th percentiles, respectively, the top whisker the largest value that is no further than 1.5 Interquartile range (IQR) from the 75th percentile, the bottom whisker the smallest value no more than 1.5IQR from the 25th percentile, and points indicate outliers. **c)** Change in accuracy

as a function of the size of the image used for non-rigid registration (left) and computation time (right). The y-axis shows how much the distance between registered landmarks changed with increasing image size (and therefore computation time), when compared to the results using the default parameters. **d)** Number of "good" matches between 4 serially sliced H&E images in N=26 samples, given different combinations of feature detectors (columns) and feature descriptors (rows). Empty boxes (white) indicate incompatible feature descriptor/detector pairs. This experiment shows that the BRISK/VGG combination consistently found the largest number of good matches, which is why they were selected as the defaults. **e)** Experiment testing image sorting. The left image shows the 40 serially sliced H&E images randomly ordered. The right panel shows the slides after being sorted by optimally ordering the leaves of a hierarchically clustered image feature distance matrix, with the dendrogram shows the ordered tree. Source data are provided as a Source Data file [1].

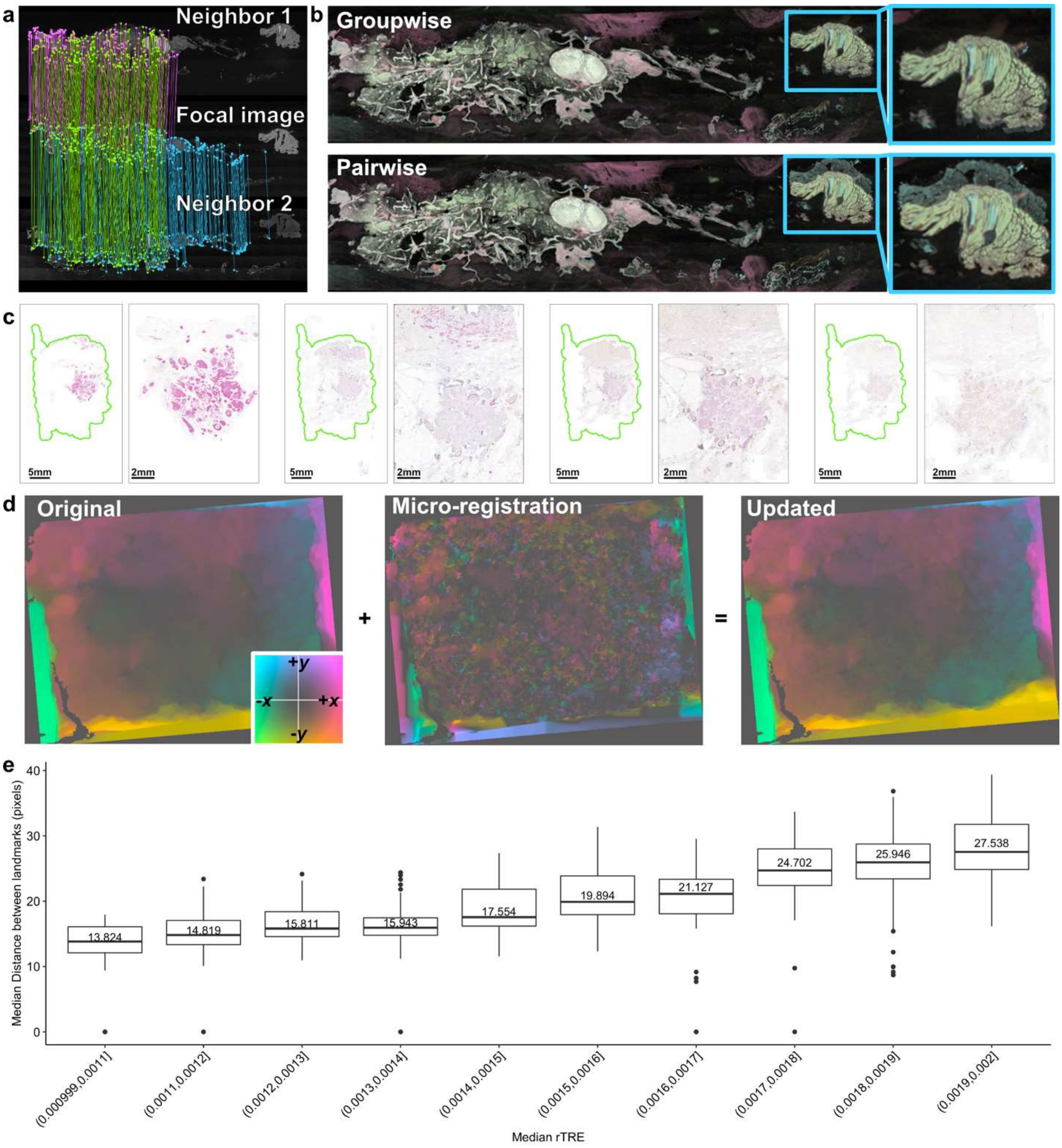

Supplemental Figure 3 Additional visualization of VALIS' pipeline and benchmarking a) Example of neighbor match filtering. Pink lines indicate features found in the image and neighbor 1 but not neighbor 2; blue lines show features found only in the image and neighbor 2; green lines show features found in all 3 images, representing those used to find the rigid transform. **b)** Example showing how serial groupwise registration can bring distant features together. In the "groupwise" panel, images were aligned serially towards the center of the stack. As transformations accumulate, the distant images have their features moved towards the center image. In contrast, when conducting direct pairwise registration, the top right piece of the tissue was too far displaced for it to be moved to the correct location. **c)** Rigid registration is performed using the entire image (left portion of image pair), while non-rigid and micro-registration is performed using higher resolution images contained within the image mask (outlined in green), as shown in the right part of each image pair. **d)** Micro-registration is performed by scaling the original displacements (left) for a larger image, using them to warp the larger image, and then non-rigidly registering those larger images. This produces new displacement fields that align the micro-features found in the higher resolution images (middle). The micro-registration displacement fields are added to the original scaled displacement fields to get a new displacement field for the larger image (right). In each displacement field image, the hue indicates the direction of the displacement, while the luminosity represents the magnitude of the displacement. Colors are relative for each displacement field. **e)** Relationship between median rTRE and registered landmark distance for rTRE between 0.001 and 0.002 using N=230 unique image pairs from the ANHIR Grand Challenge dataset. Text indicates the median distance between registered landmarks for each rTRE range. In each box, the center line indicates the median, the top and bottom indicate the 75th and 25th percentiles, respectively, the top whisker the largest value that is no further than 1.5 Interquartile range (IQR) from the 75th percentile, the bottom whisker the smallest value no more than 1.5IQR from the 25th percentile, and points indicate outliers.

Example spatial analyses

Estimated alignment error was low for samples that underwent cyclic immunofluorescence (CyCIF), with an average distance between matched features (in the full resolution slide) being 2-6 µm apart. In these cases, the quality of the image registration was high enough that cell segmentation and phenotyping could be performed, as shown in Figures 6a and Supplementary Figure 7b.

More detailed examples illustrate how spatial analyses can be performed using multiplexed images created by merging registered images, when the registration is (CyCIF) and is not (IHC) accurate enough for cell segmentation. Registration performed on CyCIF images was highly accurate, with an alignment error less than 10µm, which is about 1 cell diameter (Figure 5). In these cases, the registration was accurate enough that cell segmentation and phenotyping could be performed. An example of such an analysis can be found in Supplementary Figure 4a-c. HALO was used for cell segmentation and marker thresholding using the 32-channel image created by merging 11 rounds of registered CyCIF images. For a full description of the channels, please refer to Supplementary Table S1. A spatial analysis of the distribution of immune cells within the carcinoma region was conducted using 13 of the 32 markers, which were used to classify cells into one of nine cell types: helper T cells, cytotoxic T cells, regulatory T cells (Treg), natural killer (NK) T cells, active cytotoxic T cells (active CTL), memory T cells, M1 macrophages, M2 macrophages, B-cells, and tumor cells (Supplementary Figure 4b, Supplementary Table S2).

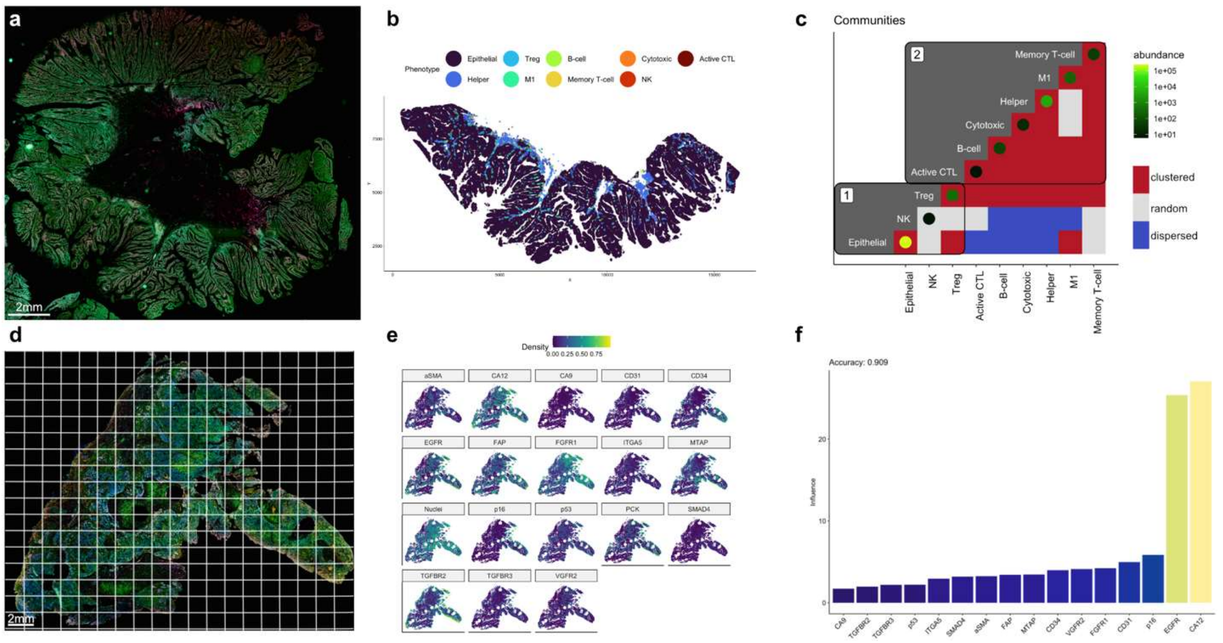

Supplemental Figure 4 Example analyses using registered WSI. **a)** A 32-channel image was created by registering and merging several rounds of CyCIF. The HALO platform was then used to perform cell segmentation and marker thresholding. **b)** Within the carcinoma region, a spatial analysis was conducted to determine the spatial relationship between 10 cell types, defined by different combinations of 13 markers. The pattern was determined using the DCLF test, where cell types could be found closer than expected (clustered), randomly distributed, or further apart than expected (dispersed). **c)** The observed patterns were used to construct a weighted network (1=clustered, 0=random, -1=dispersed), which subsequently underwent community detection. These results indicate the carcinoma (Epithelial) is largely isolated from the immune system. **d)** A composite IHC image of HNSCC using 18 markers of the tumor microenvironment. Alignment of IHC may not be cell-cell perfect, but using ecological methods, a spatial analysis can be conducted using quadrat counts. Each aligned slide underwent stain segmentation, the results of which were merged into a single composite image that was divided into regular quadrats. **e)** The number of positive pixels of each marker was calculated for each quadrat. **f)** A species distribution model was fit to the data to determine the role of each marker in creating a pro-tumor environment. Here, CA12 and EGFR were found to play the largest roles in creating a tumor supporting habitat. Source data are provided as a Source Data file[1].

A spatial analysis of the immune composition was conducted by first determining the spatial pattern observed between each pair of cell types (e.g. clustered, complete spatial randomness (CSR), or dispersion). Significance of departure from CSR was determined using the Diggle-Cressie-Loosmore-Ford (DCLF) test on the cross-type L-function for homogeneous point patterns (i.e. Besag's transformation of Ripley's K function) [2, 3, 4, 5]. These tests were conducted using the spatstat package for R [6, 7]. Clustering was considered significant when $p \leq 0.05$ for the alternative hypothesis of "greater", i.e. there were more cells within a radius *r* than expected under CSR. The spatial pattern was classified as dispersion when $p \leq 0.05$ for alternative hypothesis of "lesser". These patterns were then used to construct a weighted adjacency matrix, where 1=clustered, 0=CSR, and -1=dispersed (Supplementary Figure 3c). The matrix was then divided into communities using the Leiden community detection algorithm [8]. This analysis revealed that the tumor (in community 1) is largely isolated from immune system (community 2).

Spatial analyses can also be conducted when alignments are not close enough for cell segmentation. One approach is to first divide the image into quadrats, and then count cells and/or quantify the markers in each quadrat. One can then can select from a wide variety of methods to conduct a spatial analysis of the quadrat counts. For example, one can create spatial association networks, species distribution models, and test for complete spatial randomness [7, 9, 10].

Examples of spatial analyses of histological data with ecological methods based on quadrat counts or multiple subregions can be found in [11, 12, 13, 14]. Here, we provide a brief example using a sample that went through 18 stain/wash cycles, each time being stained for one of 18 tumor microenvironment (TME) markers (Figure 6b, Supplemental Figure 4d-f) (EGFR, H&E, FAP, α-SMA , TGFBR2, p16, FGFR1, TGFBR3, PCK, VGFR2, MTAP, CD34, CA9, p53, SMAD4, ITGA5, CA12, CD31). Each image underwent stain segmentation, the results of which were merged to create a single 18-channel composite slide (Figures 6b and Supplementary Figure 4d). This slide was then divided into 100μm x 100μm quadrats, and the number of positive pixels per quadrat for each marker was recorded (Supplementary Figure 4a, b). Species distribution models (SDM) are used by ecologists to quantify the importance of environmental factors (including other species) in creating niche that supports a species of interest [15]. Here, we fit an SDM to the quadrat counts in order to quantify the importance of each marker in creating a hospitable tumor microenvironment (Supplementary Figure 4c). The results from this analysis indicate that EGFR and CA12 play the largest role in creating a pro-tumor microenvironment.

**CyCIF Round**	**Non-DAPI Channels**
Round 1	pHH3
	iNOS
	CD45
Round 2	CD163
	CK
	CD8
Round 3	CD44
	HLA-DR
	PD-1
Round 4	Ecad

	CD3
	CD20
Round 5	CD4
	γ-H2AX
	PD-L1
Round 6	CD45RO
	FoxP3
	α-SMA
Round 7	p53
	CD68
	CD31
Round 8	CD11b
	IDO1
	Vista
Round 9	β-catenin
	HLAABC
	Myelo
Round 10	CD45RB
	Ki67
	CD57
Round 11	CD163
	CK
	Vimentin

Supplemental Table 1 Markers per registered CyCIF round. In addition to the markers lists, each round also had DAPI channel, which was used to register the rounds.

Phenotype	Marker(s)
helper T cells	CD3, CD4
cytotoxic T cells	CD3, CD8
regulatory T cells (Treg)	CD3, FOXP3
natural killer (NK) T cells	CD3, DD57
active cytotoxic T cells (active CTL)	CD3, CD8, HLA-DR
memory T cells	CD3, CD8, CD45RO
M1 macrophages	CD68, iNOS
M2 macrophages	CD68, CD163
B-cells	CD20
tumor cells	CK and/or E-cadherin

Supplemental Table 2 Cell phenotypes, and the makers used to define those phenotypes, used in the example spatial analysis using registered CyCIF rounds (Figures 5b, 6b-c).

Marker
MTAP
CD34
ITGA5
p53
p16
FAP
EGFR
TGFBR2

TGFBR3
SMAD4
FGFR1
PanCK
CA9
aSMA
VGFR2
CD31
CA12

Supplemental Table 3 Markers used in example spatial analysis of registered IHC images (Figures 5b, 6d-f).

References

1. Gatenbee C. Source data for VALIS: Virtual Alignment of pathoLogy Image Series for multi-gigapixel whole slide images publication.) (2023).

2. Diggle PJ. Displaced amacrine cells in the retina of a rabbit: analysis of a bivariate spatial point pattern. *Journal of Neuroscience Methods* **18**, 115-125 (1986).

3. Ripley BD. Modelling Spatial Patterns. *Journal of the Royal Statistical Society Series B (Methodological)* **39**, 172-212 (1977).

4. Ripley BD. *Spatial Statistics*. John Wiley & Sons (1981).

5. Besag J. Discussion of Dr Ripley's paper. *Journal of the Royal Statistical Society Series B (Methodological)* **39**, 193-195 (1977).

6. R Core Team. R: A Language and Environment for Statistical Computing.). R Foundation for Statistical Computing (2019).

7. Baddeley A, Rubak E, Turner R. *Spatial Point Patterns: Methodology and Applications with R*. Chapman and Hall/CRC Press (2015).

8. Traag VA, Waltman L, van Eck NJ. From Louvain to Leiden: guaranteeing well-connected communities. *Sci Rep* **9**, 5233 (2019).

9. Popovic GC, Warton DI, Thomson FJ, Hui FKC, Moles AT. Untangling direct species associations from indirect mediator species effects with graphical models. *Methods in Ecology and Evolution* **10**, 1571-1583 (2019).

10. Hijmans RJ, Phillips S, Leathwick J, Elith J. dismo: Species Distribution Modeling.) (2017).

11. Gatenbee CD, Minor ES, Slebos RJC, Chung CH, Anderson ARA. Histoecology: Applying Ecological Principles and Approaches to Describe and Predict Tumor Ecosystem Dynamics Across Space and Time. *Cancer Control* **27**, 1073274820946804 (2020).

12. Maley CC, Koelble K, Natrajan R, Aktipis A, Yuan Y. An ecological measure of immune-cancer colocalization as a prognostic factor for breast cancer. *Breast Cancer Res* **17**, 131 (2015).

13. Hunter MV, Moncada R, Weiss JM, Yanai I, White RM. Spatially resolved transcriptomics reveals the architecture of the tumor-microenvironment interface. *Nature Communications* **12**, 6278 (2021).

14. Gatenbee CD*, et al.* Immunosuppressive niche engineering at the onset of human colorectal cancer. *Nat Commun* **13**, 1798 (2022).

15. Hijmans RJ, Phillips S, Leathwick J, Elit J. dismo: Species Distribution Modeling. (2017).
